# Microbial Diversity in Groundwater and Its Response to Seawater Intrusion in Beihai City, Southern China

**DOI:** 10.3389/fmicb.2022.876665

**Published:** 2022-07-13

**Authors:** Zhonglin Ma, Long Gao, Mingxue Sun, Yongjie Liao, Shijie Bai, Zijun Wu, Jiangtao Li

**Affiliations:** ^1^State Key Laboratory of Marine Geology, Tongji University, Shanghai, China; ^2^Marine Geological Survey Institute of Guangxi Zhuang Autonomous Region, Beihai, China; ^3^Institute of Deep-Sea Science and Engineering, Chinese Academy of Sciences, Sanya, China

**Keywords:** seawater intrusion, Beihai City, groundwater, microbial diversity, aquifers

## Abstract

Seawater intrusion is a major concern commonly found in coastal aquifers worldwide. Because of the intense aquifer exploitation and land-based marine aquaculture in the coastal area of Beihai City, Guangxi Zhuang Autonomous Region, China, numerous underground aquifers in this area have been affected by seawater intrusion. However, the microbial communities in freshwater aquifers and their response to seawater intrusion are still unclear. In this study, groundwater from three aquifers was collected from three monitoring sites at different distances from the coastline in the coastal area of Beihai City, and the hydrochemical characteristics of these groundwater samples and the structure of the associated microbial communities were analyzed. The Cl^−^ concentration of the samples indicated that seawater intrusion had occurred in the research area up to 1.5 km away from the coastline, but the monitoring site 2 km away from the coastline had yet to be affected. Statistical analysis showed that the bacterial communities in different groundwater aquifers were significantly correlated with the Cl^−^ concentration, thereby suggesting that the extent of seawater intrusion might be one of the primary factors shaping bacterial composition in groundwater of this area, but the composition and distribution of archaea did not show a significant response to seawater intrusion and presented no apparent correlation with the Cl^−^ concentration. α-, γ-Proteobacteria and Bacteroidota were the dominant bacterial lineages, accounting for about 58–95% of the bacterial communities. Meanwhile, the predominant archaeal taxa were mainly composed of Crenarchaeota, Nanoarchaeota, and Thermoplasmatota, as accounting for 83–100%. Moreover, there was significant spatial heterogeneity of microbial communities in the aquifers affected by varying degrees of seawater intrusion. The microbial communities inhabiting the unconfined aquifer were influenced by the geochemical fluctuation caused by seawater infiltration from land-based marine aquaculture ponds and the diffusion of eutrophic surface water. In contrast, changes in microbial community structure in the confined aquifers were closely related to the environmental gradient caused by different degrees of seawater intrusion. In addition, we also found that the tidal cycle did not significantly affect the structure of microbial communities inhabiting confined aquifers that had been long affected by seawater intrusion.

## Introduction

Seawater intrusion is a global issue and has becoming increasingly serious due to the over-exploitation of groundwater caused by the increasing demand for freshwater and from the rising sea level resulting from global warming (Qahman et al., 2005; Werner et al., 2013). At present, more than 100 countries and regions in the world are threatened or affected by seawater intrusion (Barlow and Reichard, 2010; Unno et al., 2015; Scharping et al., 2018; Adyasari et al., 2019). Seawater intrusion can lead to the salinization of groundwater, resulting in a substantial reduction in the availability of potable, irrigation and industrial waters (Abd-Elhamid and Javadi, 2011; Han et al., 2011). Microorganisms are an important part of the groundwater ecosystem as they play a crucial role in the transport of nutrients, biogeochemical cycling of elements and degradation of contaminants (Sang et al., 2018). The distribution of microorganisms in groundwater is closely related to the physicochemical properties of groundwater. This is especially true in the saltwater-freshwater transition zone formed by seawater intrusion, where the physicochemical features of groundwater are altered due to seawater mixing and therefore, the diversity and structure of the microbial communities inhabiting this zone also change accordingly (Hery et al., 2014; Unno et al., 2015; Adyasari et al., 2019; Chen et al., 2019a). Environmental factors such as Cl^−^ concentration, dissolved oxygen, organic carbon, CO_2_ partial pressure, and oxidation–reduction potential play a crucial role in shaping microbial communities in the saltwater-freshwater mixing zone (Hery et al., 2014; Chen et al., 2019a). Some microbial lineages in the salt-freshwater transition zones display good correlation with the above mentioned environmental parameters. For example, in a carbonate coastal aquifer of Spain, the proportions of the Gammaproteobacteria increased with salinity along a salinity gradient, while several bacterial orders including the Desulfobacterales, Desulfovibrionales, Campylobacterales, and Alteromonadales were negatively correlated with CO_2_ partial pressure (Hery et al., 2014). Several recent investigations have revealed that microbial community structures of groundwater shift remarkably with the degree of seawater intrusion (Unno et al., 2015; Adyasari et al., 2019; Chen et al., 2019a). In addition, hydrological conditions, such as discharge rate, groundwater level and tide, can also have a significant influence on the composition of microbial communities in underground aquifers (Lee et al., 2017)., Which in turn, affects the geochemical cycling of C, N, S and Fe (Santoro et al., 2006; McAllister et al., 2015). However, on the global scale, due to the different degrees of seawater intrusion in different regions and the large differences in regional hydrogeological conditions, the microbial communities inhabiting groundwater and the influence of seawater intrusion on them are still unclear.

Beihai City, Guangxi Zhuang Autonomous Region, China, is located on the northeast coast of the Beibu Gulf (located in the north-western South China Sea). It is a peninsula city surrounded by the sea on the west, east, and south. Rivers are rare and short, and the quality of river water has been affected by industrial use, so groundwater became the primary source of drinking and agricultural water. Between 1989 and 1993, seawater intrusion was first discovered along the northwest coast of the Haicheng District of Beihai City (Zhou et al., 2000a). Subsequently, obvious seawater intrusion in other coastal areas of Beihai City was also detected. Studies showed that intense aquifer exploitation and land-based marine aquaculture were the main causes of seawater intrusion and subsequent salinization of groundwater in Beihai City (Zhou et al., 2000a; Li et al., 2018). Although a series of studies have been carried out on seawater intrusion in Beihai City, these studies were primarily based on hydrochemical and isotopic methods, focusing on the impact of seawater intrusion on groundwater salinization and investigating the degree of seawater intrusion. As far as we know, no relevant research has been conducted on the microorganisms inhabiting the groundwater and their response to seawater intrusion. Therefore, the microbial community structure in groundwater affected by seawater intrusion in the southern coastal area of Beihai City was systematically investigated in this work. Our results indicate that the varying extents of seawater intrusion and the resulting environmental gradients were the primary factors affecting the diversity and structure of microbial communities in the groundwater of Beihai City.

## Materials and Methods

### Regional Geology Introduction

**Figure 1:**
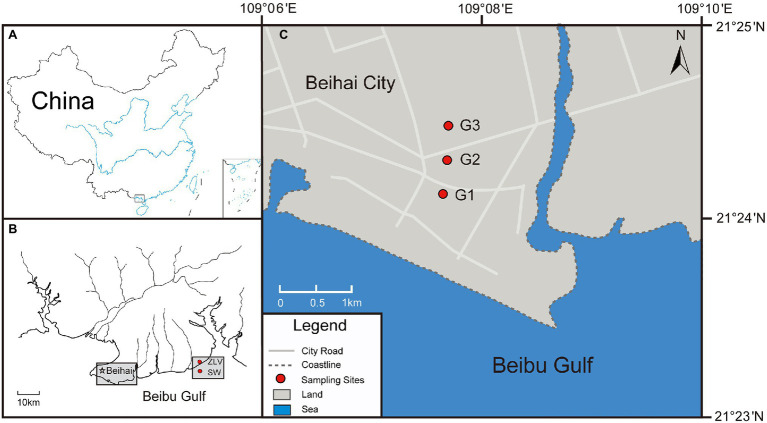
Schematic diagram of the study area and sampling location. **(A)** The black rectangle in the lower part of the plot indicates the location of Beihai City. **(B)** The southern coastal area of Beihai City and the location of the three monitoring sites involved in this study, the location of seawater (SW) collected from the Beibu Gulf and the location of groundwater discharge site (ZLV-CI) from the Zhuoluo beach. **(C)** The location of the three monitoring sites (G1, G2 and G3) illustrated in **(B)**.

Beihai City is located on the northeast coast of the Beibu Gulf, South China Sea and is surrounded by the sea on the west, east, and south (Figure 1). This area is a coastal plain ranging in elevation from 8 to 20 m (Zhou et al., 2006). Groundwater resources mainly exist in Quaternary and Neogene sedimentary aquifers, which are primarily composed of unconsolidated sand with gravel and sandy clay. The underground aquifer in this area can be divided into four layers: an unconfined aquifer and three confined aquifers (I, II, and III, Supplementary Figure 1; Li et al., 2018). A vulnerability evaluation of the regional geological environment indicated that the topography, lithologic composition, and low groundwater level in Beihai City all made the coastal area vulnerable to seawater intrusion (Ma et al., 2019). Overexploitation lead to the continuous lowering of the groundwater level and subsequent seawater intrusion, with obvious seawater intrusion first observed in the 1980s (Zhou et al., 1997, 2000a). In addition, seawater from abandoned land-based marine aquaculture ponds, a once prevalent practice in Beihai City but since prohibited, can still leak into the groundwater, aggravating seawater intrusion into and pollution of the groundwater (Li et al., 2018).

### Sample Collection

The sampling areas were located at the beaches of the Hetang area and Zhuoluo at Huangshao Village along the southern coast of Beihai City, and their geographical coordinates were 109°9′42′ E, 21°24′29” N and 109°30′53′E, 21°29′33” N, respectively. To assess the degree of seawater intrusion, three monitoring sites, 1 km (G1), 1.5 km (G2), and 2 km (G3) from the coastline, were set up in the Hetang area (Figure 1). Each monitoring site was equipped with three monitoring wells to collect groundwater from the unconfined aquifer and confined aquifers I and II (Supplementary Figure 1).

From 18th October to 20th October 2018, groundwater samples were taken from the different aquifers at the three monitoring sites (Supplementary Figure 2) to analyze their hydrochemical characteristics and the microorganisms inhabiting them. In order to ensure that representative groundwater was collected, an electric pump was used to pump water out of the monitoring wells for approximately 20 min before each sampling so as to completely discharge the non-representative groundwater out of the wells. For hydrochemical analysis, 200 ml of groundwater was collected every hour during a complete tidal cycle on the day of sampling. The collected fresh groundwater was immediately filtered through a Ф 47 mm nitrocellulose (NC) membrane (0.45 μm nominal pore size, Millipore, United States). Then, 200 μl of ultra-pure nitric acid was added to the filtered groundwater samples, before storage in brown polyethylene bottles at 4°C until laboratory analysis. The acidified groundwater samples were diluted 50-fold with double distilled water and then Cl^−^ and SO_4_^2−^ concentrations were measured by ion chromatography (ICS-1500, Dionex, United States). Total dissolved solids (TDS) of groundwaters were measured by electrical conductivity meter (MP513, Sanxin, China).

Groundwater samples for microbiological analysis were collected at high tide from the unconfined aquifer and confined aquifers I and II at the three monitoring sites. At the same time, in order to compare the influence of the tide on microorganisms in groundwater, we also collected groundwater samples from the unconfined aquifer and confined aquifers I and II at low tide at the monitoring site (G1) closest to the shoreline. In addition, seawater samples were collected from the marine area near Zhuoluo at Huangshao Village, about 35 km away from Hetang area. There is a groundwater outlet from confined aquifer I at the beach near this seawater sampling site (Supplementary Figure 2), which is submerged by seawater at high tide and exposed on the beach at low tide, with a significant groundwater outburst. For comparative purposes, groundwater samples (ZLV-CI) from this outlet were also collected for microbiological analysis. The volume of groundwater and seawater used for microbiological studies was 10 L. After sampling, microorganisms were collected by filtering the water through a Ф 147 mm polycarbonate (PC) membrane (0.22 μm nominal pore size, Millipore, United States) using a peristaltic pump. The membranes containing microorganisms were folded into sterile 15 ml centrifuge tubes, stored on dry ice, shipped to the laboratory, and then stored at −80°C until analysis.

### DNA Extraction, Library Construction and Sequencing

DNA was extracted using the DNeasy PowerMax Soil Kit (QIAGEN, United States), following the manufacturer’s instructions. Prior to extraction, the membrane was cut into as small pieces as possible with a sterile scissor. For 16S rRNA gene amplification the primer set 515F (5′-GTG CCA GCM GCC GCG GTA A-3′)/907R (5′-CCG TCA ATT CMT TTR ADT TT-3′) was used for bacteria (Bates et al., 2011; Xia et al., 2011) targeting V4-V5 region in order to minimize the overestimation due to intragenomic heterogeneity of 16S rRNA gene (Sun et al., 2013), and the primer set 519F (′-CAG CCG CGG TAA-3′)/915R (5′-GTG CTC CCC CGC CAA TTC CT-3′) was chosen for archaea due to its good specificity for the archaeal community (Yu et al., 2020). PCR amplification was performed with an initial denaturation step of 94°C for 5 min, followed by 30 cycles of 94°C for 30 s, 52°C for 30 s, and 72°C for 30 s, after which there was a final extension step of 72°C for 10 min. Each sample was run in triplicate. Library construction was performed according to the NEBNext^®^ Ultra^™^ II DNA Library Prep Kit for Illumina^®^ (New England Biolabs, United States). We selected Illumina HiSeq 2,500 and NovaSeq 6,000 platforms to perform the paired-end sequencing for bacterial and archaeal libraries, respectively.

### Quantification of 16S rRNA Gene and Cell Abundance Estimation

The prokaryotic 16S rRNA gene was quantified by using qPCR. The primers 341F (5’-CCT ACG GGA GGC AGC AG-3′) and 518R (5′-ATT ACC GCG GCT GG-3′) were used for the 16S rRNA gene of bacteria (Shi et al., 2011), while the primer set 519F (5’-CAG CMG CCG GTA A-3′) and 806R (5′-GGA CTA CVS GGG TAT CTA AT-3′) were used for the archaea (Aromokeye et al., 2018). The PCR product of the bacterial and archaeal 16S rRNA gene was cloned into a pUC18 plasmid vector (Takara Bio, Japan), and then transferred to *Escherichia coli* DH5α (Tarkara Bio, Japan) to construct a recombinant plasmid. After extraction and purification, the recombinant plasmid was used as a DNA template and diluted 10-fold as the standard for qPCR. The *R*^2^ of the standard curves varied between 0.98 and 0.99.

qPCR was performed using a 96-well PikoREAL qPCR System (Thermo Fisher Scientific, United States). PCR was carried out in a 10 μl amplification volume, containing 1 μl of DNA template, 0.2 μl Rox Reference Dye (Takara Bio, Japan), 0.2 μl each of forward and reverse primers, 5 μl SYBR Green (Takara Bio, Japan) fluorescent dye and 3.4 μl double distilled water. The PCR conditions were as follows: firstly, 95°C for 30 s, then, 40 cycles of 95°C for 5 s, 55°C for 30 s, 72°C for 60 s, lastly, 72°C for 1 min. Each sample was run in triplicate, and the average copy number of the 16S rRNA gene was calculated. The cell abundance of bacteria and archaea was estimated based on the average copy number of the 16S rRNA gene of bacterial and archaeal cells revealed in the rrNDB database: it was assumed that each bacterial cell contained an average of 5.2 copies of the 16S rRNA gene while archaea contained 1.7 copies per cell (Stoddard et al., 2015).

### Sequence Processing and Taxonomic Annotation

Raw sequences from amplicon sequencing were processed using the QIIME2 pipeline (Version 2021.2; Bolyen et al., 2019). By using the DADA2 (Version 1.18.0) plugin in QIIME2, the sequences were first filtered, denoised, and merged, followed by removal of chimeras to obtain the Amplicon Sequence Variation (ASV) table (Supplementary Tables 1, 2) and representative sequences (default parameters; Callahan et al., 2016). To avoid errors due to the unequal number of sequences, all reads of each sample in the ASV table were normalized by resampling of sequences for each sample based on the sample with the fewest number of sequences using the q2-feature-table plugin in QIIME2. Using the resampled ASV table, α diversity indices (Chao1, Shannon, Simpson) were calculated and the β diversity was statistically analyzed using the q2-diversity plugin of QIIME2. The Naive Bayes Classifier was trained according to the amplified fragments by the Silva database (Version 138) for bacteria and archaea, respectively. Then representative sequences were annotated with the trained classifier using the q2-feature-classifier plugin of QIIME2 (Bokulich et al., 2018).

In order to analyze the microbial community composition, ASVs with relative abundance greater than 1 and 0.1% in all the samples from the ASV table of bacteria and that of archaea were selected, respectively. After aggregation, the top 10 lineages of bacteria at the phylum level (Proteobacteria at the class level) and archaea at the class or order level were selected, and the remaining taxa were grouped together as “others” (Supplementary Figure 3). To analyze community composition bar plots were drawn with the R package ggplot2 (version 3.3.5; Hadley, 2011). In addition, ASVs from the bacterial ASV table with a relative abundance greater than 3% in all the samples were selected to draw a bubble plot to analyze the bacterial community composition at finer taxonomic resolution.

### Statistical Analysis

In order to reveal the heterogeneity of the microbial composition among samples, we calculated the Weighted-UniFrac Distance Matrix in QIIME2, and then performed Non-metric Multidimensional Scaling (NMDS) analysis using R package Vegan (Version 2.5–7; Oksanen et al., 2020). The Analysis of Similarities (ANOSIM) and Permutational Multivariate Analysis of Variances (PERMANOVA) methods were also used to test the significance of the NMDS grouping results. In order to identify the major microbial groups that contributed to the differences among different groups in NMDS and the differences between high tide and low tide at the G1 monitoring site, we conducted a Similarity Percentages (SIMPER) analysis based on the Bray-Curtis distance matrix using the PAST software (Version 4.07b; Hammer et al., 2001). In addition, in order to test the potential association between microbial diversity and seawater intrusion, Pearson correlation coefficients of α diversity indices and Cl^−^ concentration were calculated and the significance of this correlation was also tested using R package ggpubr (Kassambara, 2020).

### Construction of the Phylogenetic Tree

In order to more intuitively reveal the differences of samples between high and low tide at monitoring site G1, the correlation sequences of abundant lineages (relative abundance greater than 10% in at least one sample from monitoring site G1) whose contribution to the difference between the high and low tide samples was greater than 1% in the SIMPER analysis results were selected to construct a phylogenetic tree (Bootstrap = 1,000) using the maximum likelihood method in MEGA7 (Version 7.0) software (Kumar et al., 2016), and the online tool iTOL (V5) was used for visualization of the tree (Letunic and Bork, 2021). The model to construct the phylogenetic tree was selected automatically by the software and in general, the one with the lowest Bayesian Information Criterion (BIC) was chosen.

## Results

### Hydrochemical Properties of Groundwater

**Figure 2:**
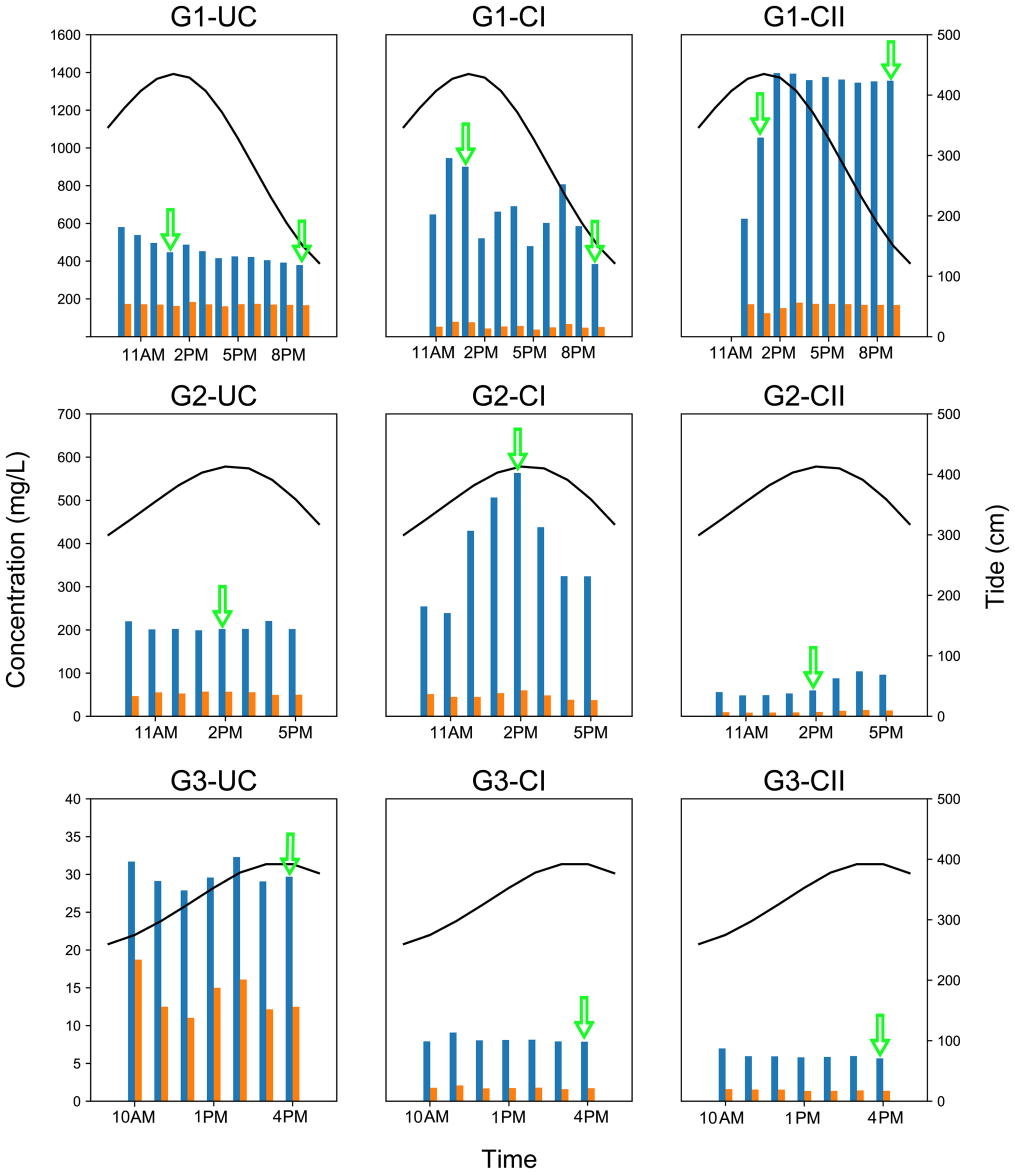
Variation of the Cl^−^ and SO_4_^2−^ concentrations and tide level in different underground aquifers of the three monitoring sites. Blue bars in the plot represent the Cl^−^ concentration and the orange bars represent the SO_4_^2−^ concentration. The black solid line above each graph indicates the tidal level of the Beihai Port of Beihai City. The green arrow indicates when microbial sampling was performed. The left y-axis represents the concentrations of Cl^−^ and SO_4_^2−^, the right y-axis represents the tidal level, and the x-axis represents time. The tidal data in the figure was obtained from the China oceanic information network (http://www.nmdis.org.cn). G1, G2 and G3 indicate monitoring site. UC, unconfined aquifer; CI, confined aquifer I; CII, confined aquifer II.

The concentrations of Cl^−^ and SO_4_^2−^ in different aquifers at three monitoring sites of varying distances from the coastline in Beihai City were analyzed. Among the three monitoring sites, the Cl^−^ concentration at site G1, closest to the coastline, was the highest (379–1,396 mg/L), the lowest concentration (about 28–32 mg/L) was at site G3, which was furthest from the coastline, and the value at site G2, 48–563 mg/L, was between the other two sites (Figure 2).

The Cl^−^ concentration in groundwater from the confined aquifers of monitoring site G1 showed the same trend with tidal levels, although with varying magnitudes. Among them, the Cl^−^ concentration of confined aquifer II reached 1,396 mg/L at high tide, and that of confined aquifer I was slightly lower at 946 mg/L. In contrast, the Cl^−^ concentration of the unconfined aquifer had the smallest variation range, 379–580 mg/L. The Cl^−^ concentration of the unconfined aquifer at monitoring site G2 had no obvious correlation with the tide, but the Cl^−^ concentration in confined aquifer I at the same site was highly consistent with the tidal change. Although the Cl^−^ concentration of confined aquifer II also showed a positive correlation with the tidal level, due to the relatively low Cl^−^ concentration, the variation was small (48–104 mg/L), and there was an obvious lag effect in the change time (Figure 2). Compared with monitoring sites G1 and G2, which were significantly affected by the tide, the Cl^−^ concentration in the aquifers at site G3 did not respond to the tidal cycle. At this monitoring site, the Cl^−^ concentration in the unconfined aquifer water was the highest (28–32 mg/L), while confined aquifers I and II were only 6–9 mg/L.

The change of the SO_4_^2−^ concentration was mostly consistent with the Cl^−^ concentration, but the concentration range (1–200 mg/L) was much smaller than that of Cl^−^ (Figure 2). The concentrations of total dissolved solids (TDS) in groundwaters also varied over a wide range from 14.6 mg/L to 6.36 × 10^3^ mg/L. In general, the groundwaters in G1 site had the highest TDS concentrations, followed by G2 site, while G3 site had the lowest TDS concentrations (Supplementary Figure 4). TDS concentration displayed a variation trend similar to that of Cl^−^ concentrations.

### Cell Abundance of Microorganisms in Groundwater

Based on the quantitative results of the 16S rRNA gene qPCR, we estimated the cell abundances of bacteria and archaea inhabiting the groundwater of Beihai City (Supplementary Table 3). Among them, the cell abundance of bacteria and archaea in the seawater collected from the sea near Zhuoluo were 2.96 × 10^10^ cells/L and 3.51 × 10^8^ cells/L, respectively. Our bacterial and archaeal estimates were higher than values obtained by other studies of coastal seawater, which generally range around the order of 10^9^ and 10^6^ ~ 10^8^ cells/L, respectively (Alderkamp et al., 2006; Lee and Bong, 2008; Carlson and Wiegner, 2016; Chen et al., 2022). This may have been related to the eutrophication of the water body in the Beibu Gulf region due to anthropogenic activity input and the high concentration of nutrient input from the Pearl River to Beibu Gulf *via* the Qiongzhou Strait (Chen et al., 2019b). The cell abundance of bacteria and archaea of ZLV-CI were 1.28 × 10^7^ cells/L and 6.00 × 10^6^ cells/L, respectively. The abundance of bacterial cells in different groundwater aquifers at three monitoring sites of the Hetang area ranged from 1.28 × 10^7^ to 8.19 × 10^10^ cells/L. In contrast, the abundance of archaea was about two orders of magnitude lower than that of bacteria, ranging from 1.66 × 10^6^ to 9.90 × 10^8^ cells/L (Supplementary Table 3).

### α Diversity Indices

See Supplementary Table 4 for the α diversity indices of bacteria and archaea inhabiting the seawater and groundwater of different aquifers at each monitoring site. In general, the unconfined aquifer had the highest diversity for bacteria or archaea among the different aquifers. Among the three monitoring sites, the microbial diversity of G1 was much lower than that of the other two, and the α diversity of G2 was slightly higher than that of G3. In addition, the α diversity of bacteria was much higher than that of archaea in most cases, with the exception that the Chao1 index of archaea was slightly higher than that of bacteria at both high tide and low tide in the unconfined aquifer at monitoring site G1 (Supplementary Table 4).

**Figure 3:**
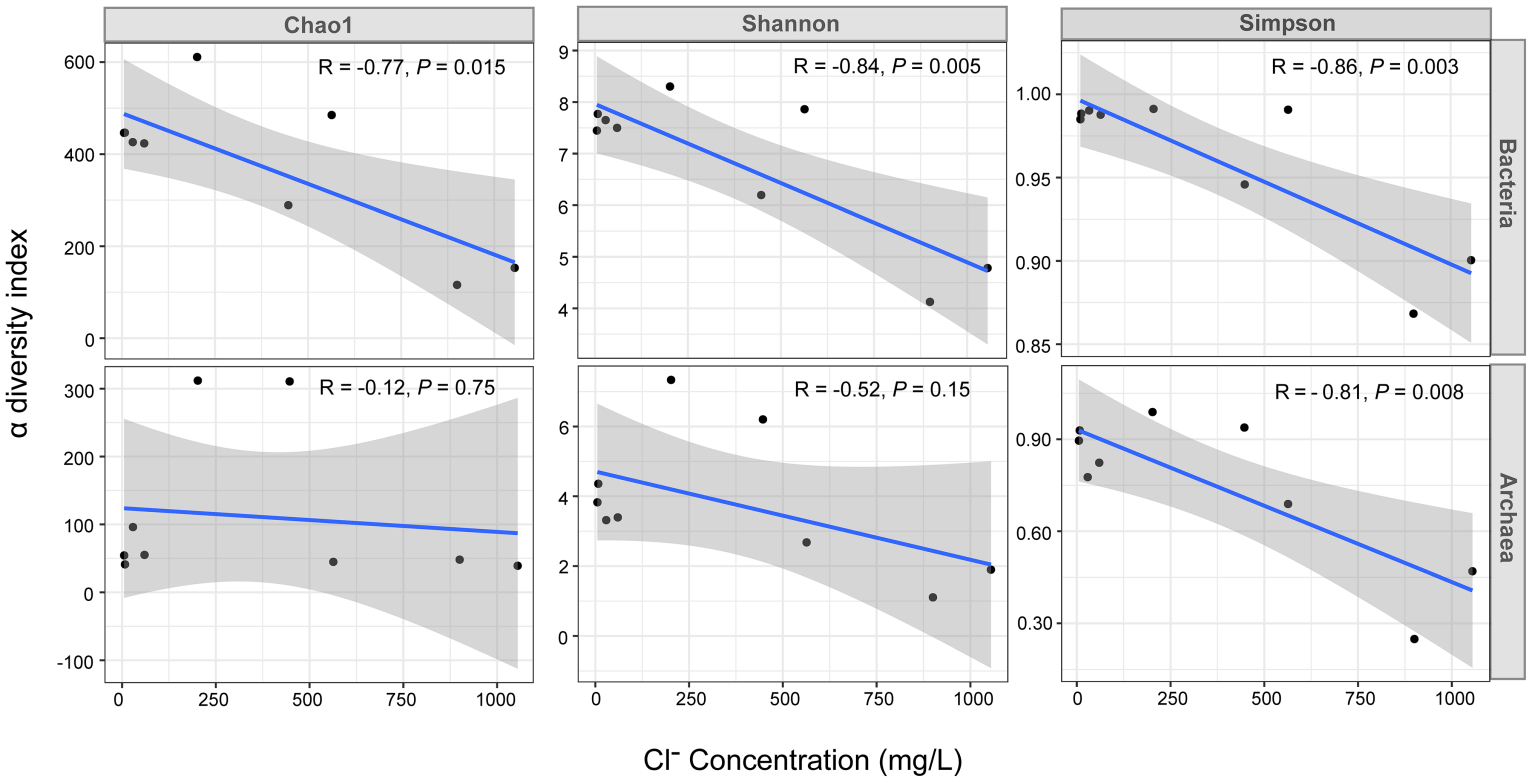
Pearson correlation analysis between the Cl^−^ concentration and the α diversity indices of bacteria and archaea. The *x*-axis represents the Cl^−^ concentration (mg/L) and the *y*-axis represents the α diversity index value. The R and value of *p* in the plot were obtained by Pearson correlation analysis, the solid blue line is the linear regression fit curve and the shaded areas represents the 95% confidence intervals. The data of nine samples including the high tide samples of G1 monitoring sites and the high tide samples of the G2 and G3 were used for this analysis.

An interesting finding was the significant negative correlation between the bacterial α diversity indices and Cl^−^ concentration (R: −0.77 to −0.86, *P*: 0.003 to 0.015). In contrast, this significant negative correlation did not exist for archaea. Only the Simpson index showed a significant negative correlation (Figure 3).

### Statistical Analysis

**Figure 4:**
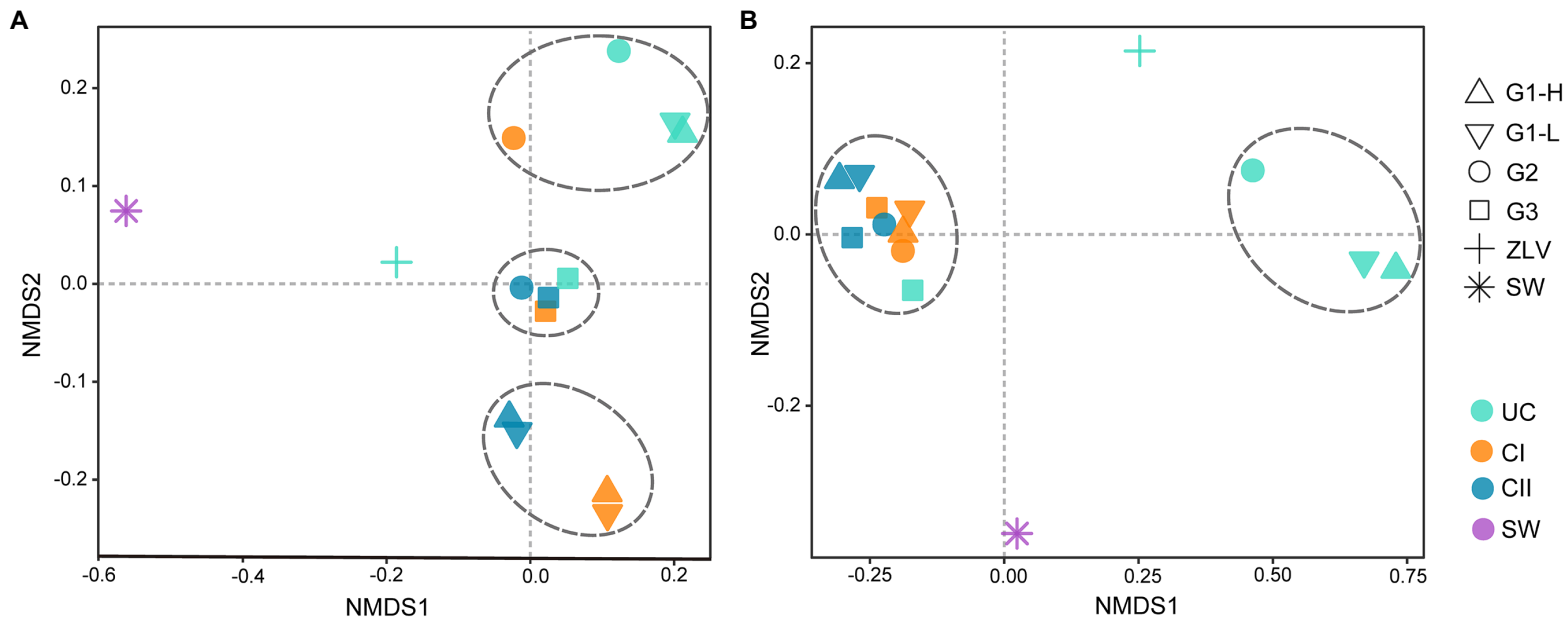
NMDS Analysis based on Weight-UniFrac distance matrix. **(A)** NMDS analysis of bacterial communities; **(B)** NMDS analysis of archaeal communities. The dashed lines divide the groundwater samples from the monitoring wells G1, G2 and G3 into different groups. Statistical analyses supported the groups with statistical significance (Supplementary Table 5). G1, G2 and G3 indicate monitoring site. ZLV, groundwater outlet at Zhuoluo; SW, seawater; UC, unconfined aquifer; CI, confined aquifer I; CII, confined aquifer II; H, high tide; L, low tide.

The bacterial NMDS analysis showed great differences in the bacterial community between seawater and ZLV-CI as well as between these two samples and the other aquifer samples (Figure 4A), indicating that the bacterial community composition of seawater was significantly different from that of groundwater. Although ZLV-CI also belonged to groundwater, it was located far away from the three monitoring sites (35 km), and ZLV-CI was exposed to air at low tide and, therefore, it could easily be affected by the local environment; thus, the bacterial community structure of the seawater and groundwater of the three groups of monitoring wells was quite different. The NMDS analysis divided the 12 samples, other than seawater and ZLV-CI, into 3 major groups (*p* < 0.05, Supplementary Table 5). The three samples of monitoring site G3 and confined aquifer II of G2 were clustered into Group 1. Combined with the Cl^−^ concentration in Figure 2, it was clear that the Cl^−^ concentrations of these four samples were the lowest among all the samples. The unconfined aquifer at G1 and G2 and confined aquifer I at G2 were clustered into Group 2, while confined aquifers I and II at G1, high and low tide, composed Group 3. These two aquifers were the most affected by seawater intrusion. The samples of high and low tide of the three aquifers at monitoring site G1 almost overlapped, indicating that there was very little difference in the bacterial composition between them at high and low tide.

The NMDS analysis of archaea also showed that there were significant differences between seawater and ZLV-CI as well as between them and the groundwater samples of the three monitoring sites (Figure 4B). Except for seawater and ZLV-CI, the other samples were mainly clustered into two groups (*p* < 0.005, Supplementary Table 5). The unconfined aquifer at G1 and G2 formed Group 1, and the remaining samples forming Group 2. The samples of high and low tide in each aquifer at G1 also clustered together, indicating that tide did not influence the composition of the archaeal community any aquifer at site G1.

SIMPER analysis showed that γ-Proteobacteria (Methylomonadaceae, Gallionellaceae, *Vogesell*, *Hydrogenophaga*, *Burkholderia-Caballeronia-Paraburkholderia*), α-Proteobacteria (Reyranellaceae, Rhodobacterales, *Novosphingobium*), Patescibacteria (Paceibacteraceae) and Bacteroidota (*Flectobacillus*) were the main lineages contributing to the clustering differences in NMDS analysis of bacterial community composition. In contrast, the differences of archaea were primarily contributed by Crenarchaeota (Nitrosotaleaceae, Nitrososphaeraceae) and Nanoarchaeota (Woesearchaeales; Supplementary Table 6).

### Community Structure of Bacteria and Archaea

**Figure 5:**
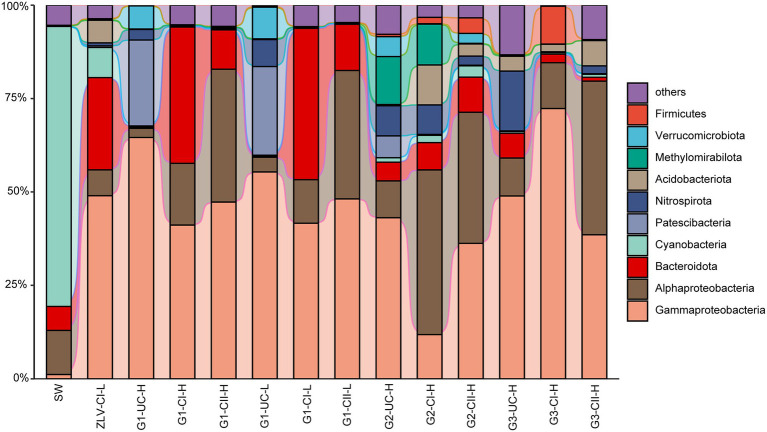
Alluvial bar plot of bacterial community composition and relative abundances. Only the top 10 classes/orders with relative abundance greater than 1% in all the samples are shown with the remaining taxa grouped together as “others” (Supplementary Figure 3A). G1, G2 and G3 indicate monitoring site. ZLV, groundwater outlet at Zhuoluo; SW, seawater; UC, unconfined aquifer; CI, confined aquifer I; CII, confined aquifer II; H, high tide; L, low tide.

**Figure 6:**
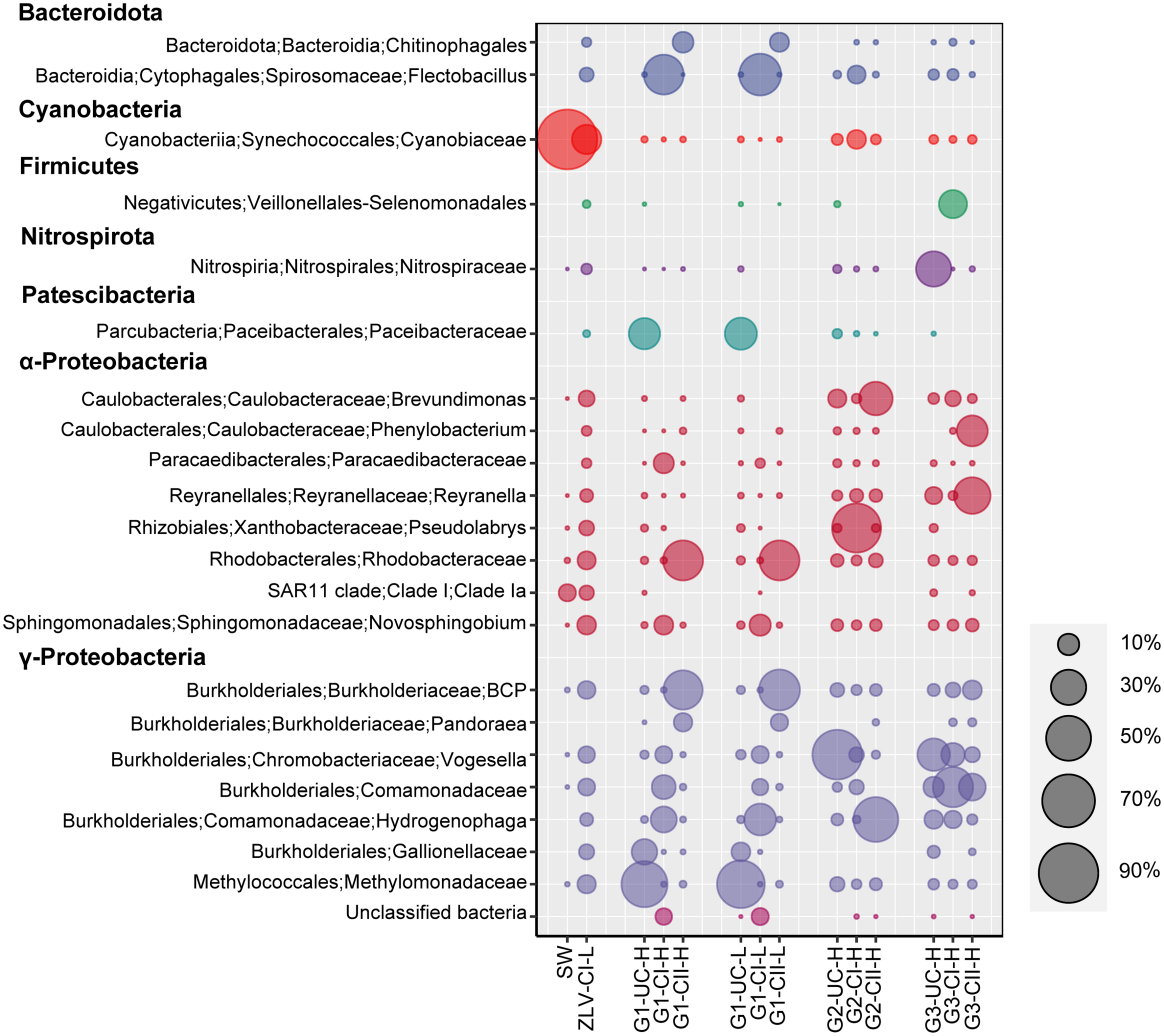
Bubble chart of the relative abundance of bacterial lineages at the genus level. The graph shows bacterial lineages with relative abundance greater than 3% in all of the samples, bubbles of different sizes represent the relative abundance of each group, and the relative abundance ratio represented by the bubble size is listed on the right side of the graph. Aggregation was performed at the genus level, some groups are annotated only to family in the Silva (138) database, so their names at genus level are not indicated in the figure. G1, G2 and G3 indicate monitoring site. ZLV, groundwater outlet at Zhuoluo; SW, seawater; UC, unconfined aquifer; CI, confined aquifer I; CII, confined aquifer II; H, high tide; L, low tide. BCP: Burkholderia-Caballeronia-Paraburkholderia.

γ-Proteobacteria, α-Proteobacteria and Bacteroidota were the most dominant bacterial groups in the groundwater samples of Beihai City, and their sum accounted for 58–95% of the bacterial community composition (Figure 5). With the exception of confined aquifer I at site G2, γ-Proteobacteria were the dominant group inhabiting the groundwater at different sites and aquifers, accounting for 36–72% of the total bacterial community. γ-Proteobacteria accounted for only 12% of the confined aquifer I at monitoring site G2, but its relative abundance in the unconfined aquifer at the G1 monitoring site and the confined aquifer I at monitoring site G3 were the highest, reaching 55–72%. Their abundance in the other groundwater was relatively stable at 36–49%. At the level of family/genus, *Burkholderia-Caballeronia-Paraburkholderia*, Gallionellaceae, Comamonadaceae, Methylomonadaceae, *Vogesella*, and *Pandoraea*, *Hydrogenophaga* were the most abundant groups within the γ-Proteobacteria (Figure 6). Their abundance and distribution were related to the monitoring sites and the aquifers they inhabited. Although α-Proteobacteria was also a dominant group in groundwater, the abundance of α-Proteobacteria varied greatly among the different monitoring sites and aquifers. In general, their relative abundance in the unconfined aquifer was low, 2–10%, but was relatively high in the confined aquifers, especially in confined aquifer II, accounting for 40–50% (Figure 5). At finer taxonomic levels, the main lineages of α-Proteobacteria included *Reyranella*, *Brevundimonas*, *Phenylobacterium*, Paracaedibacteraceae, Rhodobacteraceae, *Pseudolabrys*, and the SAR11 clade (Figure 6). Similar to the distribution of γ-Proteobacteria, the distribution of α-Proteobacteria in groundwater was closely related to the monitoring site and aquifer. Bacteroidota was another very common group in the groundwater aquifers of our research study. Their relative abundance was generally less than 12% in most cases, and as low as 1% in the unconfined aquifer at the G1 monitoring site. However, their abundance was as high as 41% in the confined aquifer I at the G1 monitoring site. In addition to the abovementioned dominant bacterial groups, some groups, such as Patescibacteria, Nitrospirota, Methylomirabilota, Firmicutes, and Acidobacteriota, had relative abundances in some groundwater samples that could reach 25%. The rare bacterial groups (ASVs < 0.01% in all the samples) were mainly composed of taxa from the Alphaproteobacteria, Planctomycetota, Chloroflexi, Verrucomicrobiota, and Bacteroidota (a more detailed taxonomic affiliation is given in Supplementary Table 7).

**Figure 7:**
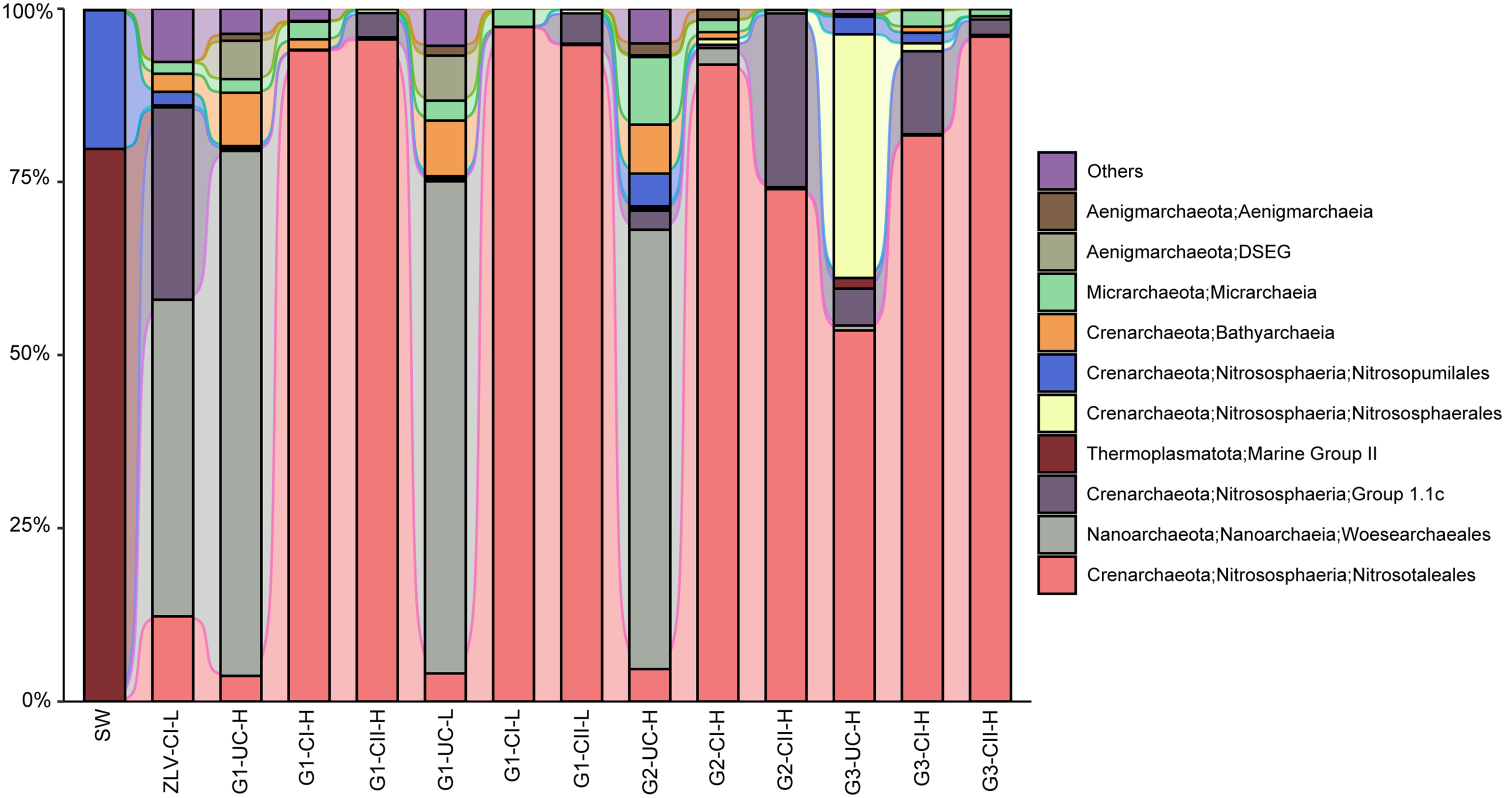
Alluvial bar plot of archaeal community composition and their relative abundance. Only the top 10 groups with a relative abundance greater than 0.1% in all the samples are shown in the figure, and the remaining taxa were grouped together as “others” (Supplementary Figure 3B). DSEG: Deep Sea Euryarchaeotic Group. G1, G2 and G3 indicate monitoring site. ZLV, groundwater outlet at Zhuoluo; SW, seawater; UC, unconfined aquifer; CI, confined aquifer I; CII, confined aquifer II; H, high tide; L, low tide.

The dominant groups of archaea in groundwater were primarily composed of Crenarchaeota, Nanoarchaeota and Thermoplasmatota, with the total abundance of these three lineages accounting for 83–100% of archaeal sequences (Figure 7). Nitrosotaleales of the Crenarchaeota, was the most dominant group of archaea, and was mainly distributed in confined aquifers I and II, reaching abundances as high as 74–97%. In contrast, they were much lower in the unconfined aquifer, typically less than 12%. The dominant archaea in the unconfined aquifer were generally of the order Woesearchaeales (Nanoarchaeota), with relative abundances reaching as high as 45–76%. In contrast, there was a significant difference between the archaeal lineages inhabiting the unconfined aquifer at monitoring site G3, and in the other two monitoring sites, the abundance of Nitrosotaleales was as high as 54%, while that of Woesearchaeales, which was absolutely dominant in the unconfined aquifer at monitoring sites G1 and G2, accounted for less than 1% (Figure 7). Another characteristic of the composition of archaea in G3 was that the order Nitrososphaerales, of the Crenarchaeota, made up a very high proportion (35%), while its relative abundance in other samples was negligible. Group 1.1c of Crenarchaeota was also a relatively abundant group, with a relative abundance of 12–28% in the groundwater discharge area (ZLV-CI), confined aquifer II at monitoring site G2 and confined aquifer I at monitoring site G3, but very little (<6%) in other samples (Figure 7). In addition, other archaeal lineages, including Bathyarchaeia and Micrarchaeota of the Crenarchaeota, and Aenigmarchaeia of the Aenigmarchaeota, were occasionally distributed in different groundwater samples, but their relative abundances were generally less than 10%. The rare archaeal ASVs (<0.01% in all the samples) mainly fell into Crenarchaeota (Bathyarchaeia, Nitrosotaleaceae, Nitrososphaeraceae, Nitrosopumilaceae) Nanoarchaeota (Woesearchaeales), Micrarchaeota, Halobacterota (Methanoperedenaceae and Halococcaceae) and Thermoplasmatota (Marine Group II; Supplementary Table 8).

Whether bacteria or archaea, the microbial composition in seawater was significantly different from groundwater. Cyanobacteria was the most important bacterial group in seawater, with a relative abundance of 75% (Figures 5, 6). Marine Group II of Thermoplasmatota was the dominant group of archaea, with an abundance of nearly 80%, followed by the order Nitrosopumilales of the Crenarchaeota, with an abundance of 20% (Figure 7). These microbial lineages were very low (<5%) in the groundwater samples. An integrated table of their main microbial components in different groundwater aquifers is available in Supplementary Table 9.

## Discussion

### Extent of Seawater Intrusion in Beihai City

The concentration of Cl^−^ in normal groundwater in Beihai City was relatively stable and generally less than 50 mg/L. In contrast, the Cl^−^ concentration in seawater is very high, and seawater intrusion will significantly increase the Cl^−^ concentration in groundwater. Therefore, the Cl^−^ concentration of groundwater is usually used as the indicator for monitoring seawater intrusion in Beihai City. When the Cl^−^ concentration of groundwater is higher than 50 mg/L, it is determined that the groundwater is suffering from seawater intrusion (Zhou et al., 1997).

The Cl^−^ concentration of groundwater in the three monitoring wells at monitoring site G1 was much higher than 50 mg/L, thereby indicating that the groundwater at this site had been invaded by seawater. The coastal area of Beihai has gentle topography, the lithologic composition is Quaternary unconsolidated sand with gravel and sandy clay, the confined aquifers and their top aquitards extend laterally to the bottom of the sea, providing a good channel for seawater intrusion (Zhou et al., 2000b; Ma et al., 2019). Because monitoring site G1 is only 1 km from the coastline, confined aquifers I and II at this site were greatly affected by seawater intrusion. The salinization of the unconfined aquifer is primarily due to the influence of nearby land-based marine aquaculture ponds, that were once prevalent in Beihai City but are now prohibited, therefore, many salinized ponds still remain on the ground. Due to the unconsolidated sedimentary formation in this area and the lack of effective anti-seepage measures along the bottoms of these culture ponds (Wu et al., 2021), the unconfined aquifer is easily salinized due to leakage of pond water. In addition, although the unconfined aquifer is higher than sea level (Supplementary Figure 1), the confined aquifer, which is salinized by seawater, will also invade the unconfined aquifer directly due to the good permeability between the unconfined aquifer and the confined aquifer (Li et al., 2018), resulting in further salinization of the unconfined aquifer. The Cl^−^ concentration results show that the Cl^−^ concentrations of the unconfined aquifer and the confined aquifer I are >50 mg/L at the G2 monitoring site, whether at the high or low tide, indicating that they have been salinized under the influence of seawater intrusion for an extended period of time. In contrast, the Cl^−^ concentration of confined aquifer II is closely related to the change of tidal level, which can be as high as 104 mg/L at high tide but can drop to 48 mg/L at low tide, which is comparable to that of normal groundwater, indicating that at the G2 monitoring site, seawater can only intrude near to the confined aquifer at the high tide, but during the ebb tide the groundwater is discharged from the confined aquifer into the sea, and under the dilution effect of groundwater, the Cl^−^ concentration at this site is reduced to below 50 mg/L, displaying the hydrochemical characteristics of underground freshwater. In general, the degree of groundwater salinization in the three aquifers of monitoring site G2 is: confined aquifer I > unconfined aquifer > confined aquifer II. At monitoring site G3, the Cl^−^ concentrations of all three groundwater aquifers were less than 50 mg/L, indicating that they were not affected by seawater intrusion at present, and this may be related to the distance of the site from the coastline, which is about 2 km. Among the three aquifers of G3, the Cl^−^ concentration of the unconfined aquifer was relatively high and stable, reaching 35 mg/L. The unconfined aquifer at this site is mainly affected by surface water, especially wastewater generated from agricultural and industrial activities (Guo et al., 2020). The microbial community structure also provides indirect evidence for this. Within the microbial community of the unconfined aquifer of the G3 monitoring site, Nitrospiraceae (bacteria) and Nitrososphaerales (archaea), which belong to typical ammonia-oxidizing microbial groups, reached relative abundances greater than 30% (Figures 6, 7). In contrast, their abundances in the unconfined aquifer at the other two monitoring sites were very low, which may be related to the higher concentration of NH_4_^+^ in the wastewater discharged from anthropogenic activities at G3.

### Influence of Tidal Cycle on the Community Structure of the Microorganisms Inhabiting Salinized Groundwater

The analysis of the microbial community structure and NMDS showed that tidal cycle had no significant influence on the microbial community inhabiting the groundwater of the three aquifers at the G1 monitoring site (Figure 4). As mentioned previously, the G1 monitoring site, has been affected by seawater intrusion at both high and low tide for an extended period of time, and the groundwater aquifers, including the unconfined aquifer, have been significantly salinized. Unlike the way in which confined aquifers I and II are salinized due to their connection with the seawater, the unconfined aquifer is higher than sea level, and salinization was caused by the early land-based marine aquaculture and the upward flow of the confined aquifers I and II that had been affected by seawater intrusion. Therefore, on the whole, the microorganism groups in the unconfined aquifer are relatively stable, and there is no direct connection with the tide, and as such the main influencing factors are surface water and precipitation (Li et al., 2018).

Confined aquifers I and II at the G1 monitoring site are directly connected to seawater (Supplementary Figure 1), such that they are directly impacted by tidal change. The incoming seawater tide will lead to seawater intrusion, and then their degree of salinization will be higher, which can be directly observed from the Cl^−^ concentration. Most studies indicate that the tidal fluctuation of seawater will alter the water chemistry properties of groundwater invaded by seawater, thus leading to corresponding changes in the microbial communities inhabiting therein and the main biogeochemical reactions associated with those communities (Grossart et al., 2004; Lee et al., 2017). However, there was no significant difference in microorganisms between high and low tide in the confined aquifers I and II of our study area, only the abundance of individual dominant groups changed slightly (Supplementary Figure 5) and SIMPER analysis (Supplementary Table 10) revealed that these microbial groups contributed the difference between high and low tides. The G1 monitoring site is 1 km from the shoreline and differs from the SGD site in the studies mentioned above as those sites very close to the coastline or even in the seawater itself. As distance from the sea increases, the effect of tides on groundwater diminishes. At low and high tide, the minimum and maximum Cl^−^ concentrations were 624.5 mg/L and 1396.0 mg/L (Figure 2), respectively, which correspond to 2.4‰ and 5.4‰ in salinity. Since monitoring site G1 had been affected by seawater intrusion for a long time, compared with freshwater, the groundwater here had been significantly salinized, and the main biological groups present had halophilic characteristics. The salinity change caused by the high and low tides was only 3‰, which is not enough to cause a significant shift in the microbial community composition (Lee et al., 2017). In addition, although the discharge of groundwater to the sea at low tide results in the introduction of microorganisms from the upstream groundwater that has not been intruded by seawater, these freshwater-adapted microorganisms find it difficult to survive in site G1 due to the high salinity (Goldscheider et al., 2006). In addition, some studies have indicated that the tidal period is too short for microorganisms to develop new microbial communities during the ebb tide stage (Lee et al., 2017).

### Impact of Seawater Intrusion on Microorganisms in the Groundwater in Beihai City

#### Relationship Between Microbial Diversity and Salinity

There was a significant negative correlation between the bacterial α diversity indices and the Cl^−^ concentration, suggesting that seawater intrusion reduced bacterial diversity in the groundwater of Beihai City (Figure 3). On the other hand, NMDS statistical analysis also found that the bacterial community structure in different aquifers was clustered roughly according to the Cl^−^ concentration (Figure 4). These results indicate that the degree of seawater intrusion is one of the primary factors shaping the bacterial community structure in different aquifers. This may be related to the fact that most bacterial groups inhabiting these aquifers tend to live in freshwater with low salinity (Lefort and Gasol, 2013; Sang et al., 2018). In contrast, the α diversity indices of archaea were not well correlated with Cl^−^ concentration, indicating that the influence of seawater intrusion on archaea was very limited (Figures 3, 4). In the aquifers of the study area, especially confined aquifers I and II which were significantly affected by seawater intrusion, the content of ammonia-oxidizing archaea (AOA) Nitrososphaeria could reach 94–99% (Figure 7). According to a previous study, such AOA generally have a relatively wide salinity tolerance range, extending from 0.47‰ to 33.3‰ (Santoro et al., 2008), indicating that they are not sensitive to salinity changes, which explains to some extent why seawater intrusion had little impact on the archaea. In addition, there were obvious differences between the microbial community composition of seawater and the microbial community structure of the underground aquifers. Typical marine microbial lineages, such as Cyanobacteria and Marine Group II, inhabiting seawater were not found in the groundwater samples, indicating that the impact of seawater intrusion on groundwater microbes was not through the direct transport or diffusion of microorganisms from seawater into groundwater. Seawater intrusion likely induce an increase of cell abundance in the groundwaters. As shown in Supplementary Table 3, groundwater aquifers displayed higher cell densities in the G1 site which had been more heavily affected by seawater intrusion than those of sites G2 and G3 which are less or scarcely influenced by seawater intrusion. This can probably attribute to the input of more nutrients from surrounding eutrophic seawater.

#### Distribution of Microorganisms in the Unconfined Aquifer

There was obvious spatial heterogeneity in the microbial community of the unconfined aquifer. Because the unconfined aquifer in Beihai City is higher than sea level (Supplementary Figure 1), it is difficult for seawater to directly intrude. But salinized confined aquifers I and II may intrude into the unconfined aquifer due to the permeability of the unconsolidated formation and the boreholes used for withdrawing groundwater, resulting in the salinization of the unconfined aquifer and a corresponding increase of the Cl^−^ concentration (Li et al., 2018; Ma et al., 2019). In addition, at the location of the G1 monitoring site, the unconfined aquifer is not only affected by the salinized confined aquifers, but also by land-based marine aquaculture seawater from culture ponds that seep down to the unconfined aquifer, resulting in further salinization of this aquifer at monitoring site G1 and a high Cl^−^ concentration (379–580 mg/L, Figure 2). The differences in microorganisms that inhabit various locations of the unconfined aquifer may be due to the local environmental conditions such as surface water and soil. For example, in the unconfined aquifer at site G3, the bacterial family Nitrospiraceae and the archaeal groups of Nitrosotaleales and Nitrososphaerales, all of which belong to typical ammonia-oxidizing microorganisms and depend on NH_4_^+^ as an electron donor (Mehrani et al., 2020), were the dominant prokaryotic groups (Figures 6, 7). This may be related to the local high nutrient surface water that results from the impact of sewage discharged from human activities at the monitoring site’s location. In addition, a large number of unconfined aquifer wells (Supplementary Figure 6) are distributed near the G3 monitoring site to meet the early stage needs of drinking water, which results in the diffusion of polluted surface water to the unconfined aquifer, resulting in an increase in many inorganic nutrients, including NH_4_^+^, in the unconfined aquifer in this area.

In contrast, the microbial community composition in the unconfined aquifer at monitoring sites G1 and G2 was significantly different from that at the G3 site. The bacterial Methylomonadaceae and Paceibacteraceae, and the archaeal Woesearchaeales dominated the unconfined aquifer at the G1 monitoring site. However, their proportion at the G3 monitoring site were less than 1% (Figures 6, 7). Previous research has shown that Paceibacteraceae are widely distributed in groundwater and that their abundance is closely related to the content of organic matter in groundwater (Herrmann et al., 2019). Similarly, Woesearchaeales, as a typical heterotrophic archaeon, has a fermentation-based lifestyle. Their survival depends on organic matter in the environment and their abundance and diversity are closely related to the abundance of organic matter (Liu et al., 2018). They are more common in salt marsh environments (Huang et al., 2021), suggesting that they may be more likely to survive and thrive in a high salinity environment. In other words, the saltier unconfined groundwater at the G1 and G2 monitoring sites provides a more suitable environment for Woesearchaeales. In addition, Methylomonadaceae is a group of specific methane-oxidizing bacteria whose metabolism depends on methyl compound substrates (Cabrol et al., 2020). Early land-based culture ponds around the G1 monitoring site, which seep into the unconfined aquifer, introduce abundant organic compounds to the unconfined aquifer potentially providing nutrients for the Methylomonadaceae. Thus, due to their preference to high N nutrients and methyl organics, Nitrospiraceae, Nitrososphaerales and Methylomonadaceae can act as potential indicators of groundwater contamination by human activities (Korbel et al., 2022). In addition, metals in groundwater may also affect the structure and function of microbial communities. Gallionellaceae, a group of microaerophilic Fe-oxidizing bacteria (Jakus et al., 2021), accounted for 15.2 and 7.7% of bacteria in the unconfined aquifer during high and low tides at the G1 site, respectively. In general, fresh groundwater provides Fe^2+^ and saline water brings O_2_, which makes it possible to oxidize Fe^2+^, mediated by Gallionaceae, in their mixing zone (McAllister et al., 2015). The higher O_2_ concentration brought by seawater at high tide could facilitate this process.

#### Distribution of Microorganisms in the Confined Aquifers and Its Relationship With Seawater Intrusion

The degree of the seawater intrusion at monitoring site G1 was the most serious, while site G2 was relatively weak and site G3 was hardly affected. This provided us with a valuable opportunity to study the spatial variation of the microbial community structure for different degrees of seawater intrusion. Previous research has shown that groundwater is often rich in inorganic nutrients, including nitrate, phosphate and silicate, while seawater provides more abundant oxygen, salt, sulfate and organic matter (Archana et al., 2021). Therefore, with the different degrees of seawater intrusion, an environmental gradient was formed among G1, G2 and G3, and the corresponding microbial community structure also showed obvious spatial differences. Along the direction of seawater intrusion, several bacterial groups, including Chitinophagales, *Flectobacillus*, Rhodobacteraceae, *Novosphingobium*, *Hydrogenophaga* and *Burkholderia-Caballeronia-Paraburkholderia* decreased significantly. However, Veillonellales-Selenomonadales, *Brevundimonas*, *Phenylobacterium* and *Reyranella* showed an increasing trend (Figure 6). Chitinophagales and *Novosphingobium* are organic heterotrophic microorganisms, both of which can degrade aromatic hydrocarbons (Takeuchi et al., 2001; Kämpfer et al., 2011). *Flectobacillus* is an aerobic bacterial genus with a strict respiratory metabolism that utilizes oxygen as the terminal electron acceptor (Hwang and Cho, 2006). Rhodobacteraceae is an important branch of the α-Proteobacteria, mainly found in marine environments, but can also be found in saline lakes and soil (Pujalte et al., 2014). These groups decrease with decreasing seawater intrusion (dominant groups in sites more affected by seawater intrusion) and it is clear that these groups have high salinity tolerance and are primarily aerobic. The increase of salinity and O_2_ content caused by seawater intrusion, and even the increase of organic matter, provides suitable environmental conditions for these groups. Similar trends have been found in studies on SGD site along the coastal zone of Jeju Island, South Korea (Lee et al., 2017). The distribution of some less abundant or rare microbial lineages were also affected by seawater intrusion. For instance, members of the Actinobacteriota showed higher relative abundances in seawater and groundwater discharging area (ZLV-CI) than those of groundwaters from three monitoring wells. As shown in Supplementary Figure 3, the relative abundances of Actinobacteria gradually decreased, and actinobacterial constituents also changed from G1 to G3 site in congruence with the decrease of seawater intrusion. On the other hand, microbial groups isolated from freshwater, lake and soil environments increased significantly in the direction of seawater intrusion (dominant groups in sites less or completely unaffected by seawater intrusion). For example, most species of *Reyranella* and *Brevundimonas* which belong to the α-Proteobacteria, have been isolated from freshwater environments and soils, and do not require NaCl for growth. They are usually salinity-independent and, therefore, are more likely to grow in groundwater that is not affected by seawater intrusion (Jayasekara et al., 1999). In addition, the distribution of some groups at the G3 monitoring site may have been more affected by human activities. For example, *Phenylobacterium* utilize the xenobiotic compound chloridazon, which is an active ingredient of herbicides and is intensively used in agriculture (Lingens et al., 1985). All in all, the spatial heterogeneity of the microbial communities inhabiting the confined aquifers was the result of gradients of environmental conditions along the direction of seawater intrusion.

One notable result was the detection of SAR11 subclade Ia in the groundwater from ZLV-CI, the unconfined aquifer of G1, and the unconfined aquifer and confined aquifer II of G3 (Figure 6). The SAR11 clade are generally considered typical marine planktonic bacteria, mainly distributed in the oligotrophic sea surface. On average, they account for a third of the cells present in surface water (Morris et al., 2002; Brown et al., 2012; Giovannoni, 2017). Among the nine branches of the SAR11 clade, only subclade IIIb has been isolated from freshwater (Henson et al., 2018). It was not unexpected that marine SAR11 subclade Ia was detected in the ZLV-CI at the groundwater discharge site at the beach, as this sample was taken from a well where residual seawater microorganisms may still be present at the low tide. However, it was surprising that this clade was found in the underground aquifer at our monitoring wells, especially at monitoring site G3, which was far from the coastline and determined to be not affected by seawater intrusion. We speculate that the possible reasons include two aspects: firstly, with the exception of subclade IIIb, it was generally believed that SAR11 are marine bacteria, however a recent study indicated that marine SAR11 subclade I/II can also occur in the freshwater lake environment (Cabello-Yeves et al., 2018), which extends the scope of freshwater SAR11 clade. At the same time, this also indicates that our knowledge of SAR11 is very limited, and the environmental distribution of the SAR11 clade is wider than previously thought, this also means that the SAR11 subclade Ia group detected in this study may be autochthonous. Secondly, due to the geographical location of Beihai City, every year it is affected by tropical cyclones between June and October, and the Beibu Gulf suffers frequent storm surge, typhoon and other natural disasters. The maximum increase of the seawater level caused by a storm surge superimposed by the spring tide can reach 286 cm, which is enough to cause seawater intrusion at monitoring site G3, which is the farthest from the coastline (Chen et al., 2020). It is possible that microorganisms brought in with the seawater may remain in the water body or the pores of the clay formation. Studies have shown that extracellular DNA can persist in the environment for extended periods of time even after the microorganism has died (Nielsen et al., 2007; Carini et al., 2017), and in a freshwater lake, despite the absence of living cells, the DNA is still amplifiable by PCR for up to 13 weeks (Deere et al., 1996). The SAR11 subclade Ia detected in our groundwater samples may have expired but we were still able to amplify their DNA. According to report^1^ issued by the Guangxi Zhuang Autonomous Region Oceanic Administration, 6 tropical cyclones affected the Beibu Gulf area in 2018. The number of days with disastrous waves (wave height of no less than 2 m) along the coast were 32, and the maximum wave height was about 4 m, which occurred during the period of Super Typhoon “Mangkhut” (NO. 1822) on 17 September, and caused storm surge disasters, with the storm surge water increase exceeding 50–70 cm. Considering that the sampling time of our study was close to the time period that Super Typhoon “Mangkhut” affected the Beibu Gulf, and that the SAR11 clade distributed in both groundwater and seawater were all subclade Ia, the second explanation may be more reasonable.

It should be noted that the sampling periods in this study were limited, largely limiting the understanding of microbial responses to seawater intrusion over longer time scales. In addition to the regular tidal cycle, the spring-neap and seasonal water table oscillations would also significantly affect saltwater-freshwater mixing dynamics (Heiss and Michael, 2014). Therefore, the impact of these variations on the microbial community should be taken into consideration during future studies.

## Conclusion

The hydrochemical characteristics and microbial community structure of the groundwater affected by seawater intrusion in the coastal area of Beihai City were investigated in this work. Our results show that the degree of seawater intrusion into the groundwater decreases with increasing distance from the coastline, and the structure of the microbial communities inhabiting the unconfined aquifer and confined aquifers I and II presented significant spatial heterogeneity. The composition and distribution of microorganisms in groundwater in the coastal area of Beihai City are related to many factors such as the degree of seawater intrusion, land-based aquaculture and the eutrophication of surface water caused by human activities. With increasing seawater intrusion, there was also an increase in the content of microorganisms with high salinity tolerance, aerobic respiration, and organic-dependent heterotrophic metabolism in general, while at the same time microbial diversity decreased. In addition, tidal change can remarkably alter the salinity of salinized groundwater which has been affected by seawater intrusion for a long time near the coast, but it has an insignificant impact on the structure of the microbial communities there.

## Data Availability Statement

The datasets presented in this study can be found in online repositories. The names of the repository/repositories and accession number(s) can be found at: https://www.ncbi.nlm.nih.gov/bioproject/PRJNA801071.

## Author Contributions

JL designed the research. ZM and JL carried out the chemical and microbial experiments and wrote the manuscript with the contributions from all authors. ZM, MS, and SB processed and analyzed the data. All authors contributed to the article and approved the submitted version.

## Funding

This research has been supported by National Natural Science Foundation of China (grant no. 42072333) and Science and Technology Commission of Shanghai Municipality (grant no. 20ZR1460600).

## Conflict of Interest

The authors declare that the research was conducted in the absence of any commercial or financial relationships that could be construed as a potential conflict of interest.

## Publisher’s Note

All claims expressed in this article are solely those of the authors and do not necessarily represent those of their affiliated organizations, or those of the publisher, the editors and the reviewers. Any product that may be evaluated in this article, or claim that may be made by its manufacturer, is not guaranteed or endorsed by the publisher.

## Footnotes

^1^ http://hyj.gxzf.gov.cn/zwgk_66846/hygb_66897/hyzhgb/t7203611.shtml
