# Supplementary material for: Microbial Diversity in Groundwater and Its Response to Seawater Intrusion in Beihai City, Southern China
Source: Front Microbiol. 2022 Jul 13;13:876665. doi: 10.3389/fmicb.2022.876665 (PMC9328385; doi:10.3389/fmicb.2022.876665)
Supplement: Supplementary file 1 [file Data_Sheet_1.DOCX]

***Supplementary Materials***

**1.1 Supplementary Figures**


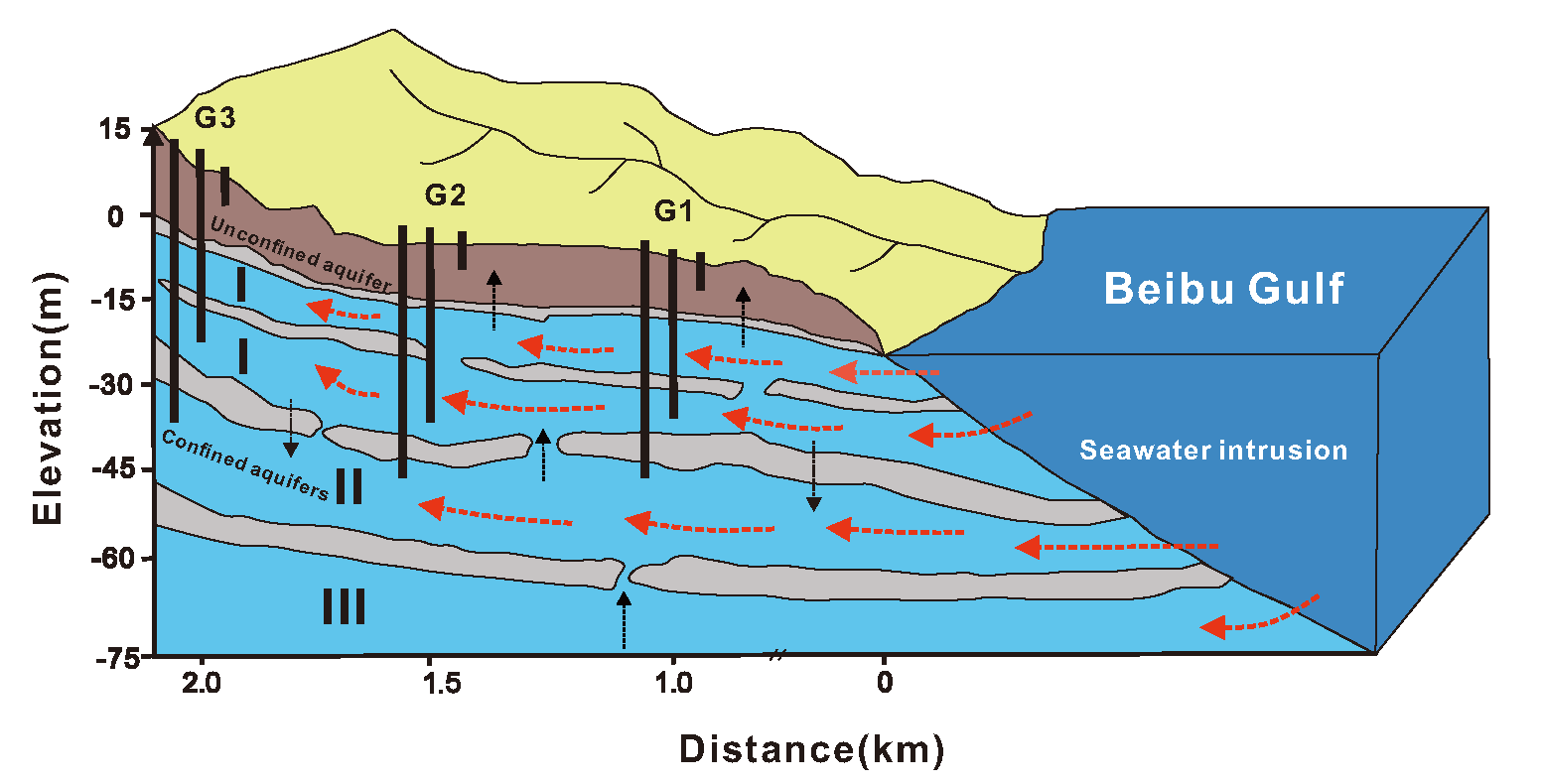


**Supplementary Figure 1**. Schematic diagram of the three groups of monitoring wells. The red arrow represents the direction of seawater intrusion, and the black dotted arrow represents the groundwater connections between aquifers. Three thick solid lines of unequal length below the name of each monitoring site indicate the wells for sampling groundwater from each aquifer. This figure was edited from Figure 11 in Li et al., 2018.


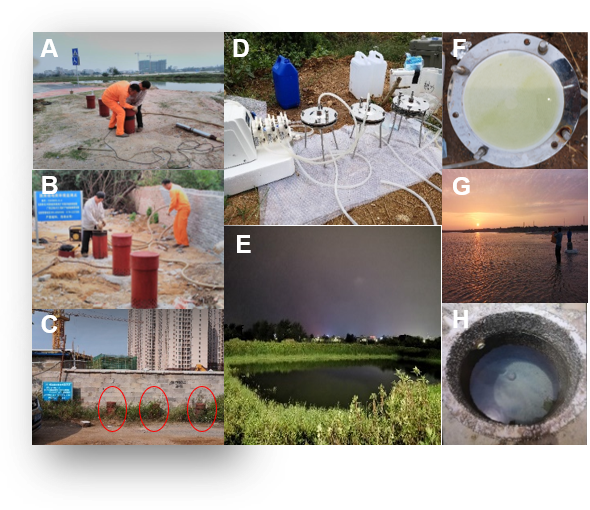


**Supplementary Figure 2**. Sampling sites. (A), (B) and (C) are monitoring sites G1, G2 and G3, respectively; (D) Groundwater sample filtration; (E) Abandoned land-based sea farming culture ponds near site G1; (F) Filter membrane; (G) Seawater sampling site; (H) Groundwater outlet from the confined aquifer I near the seawater sampling site (ZLV-CI).


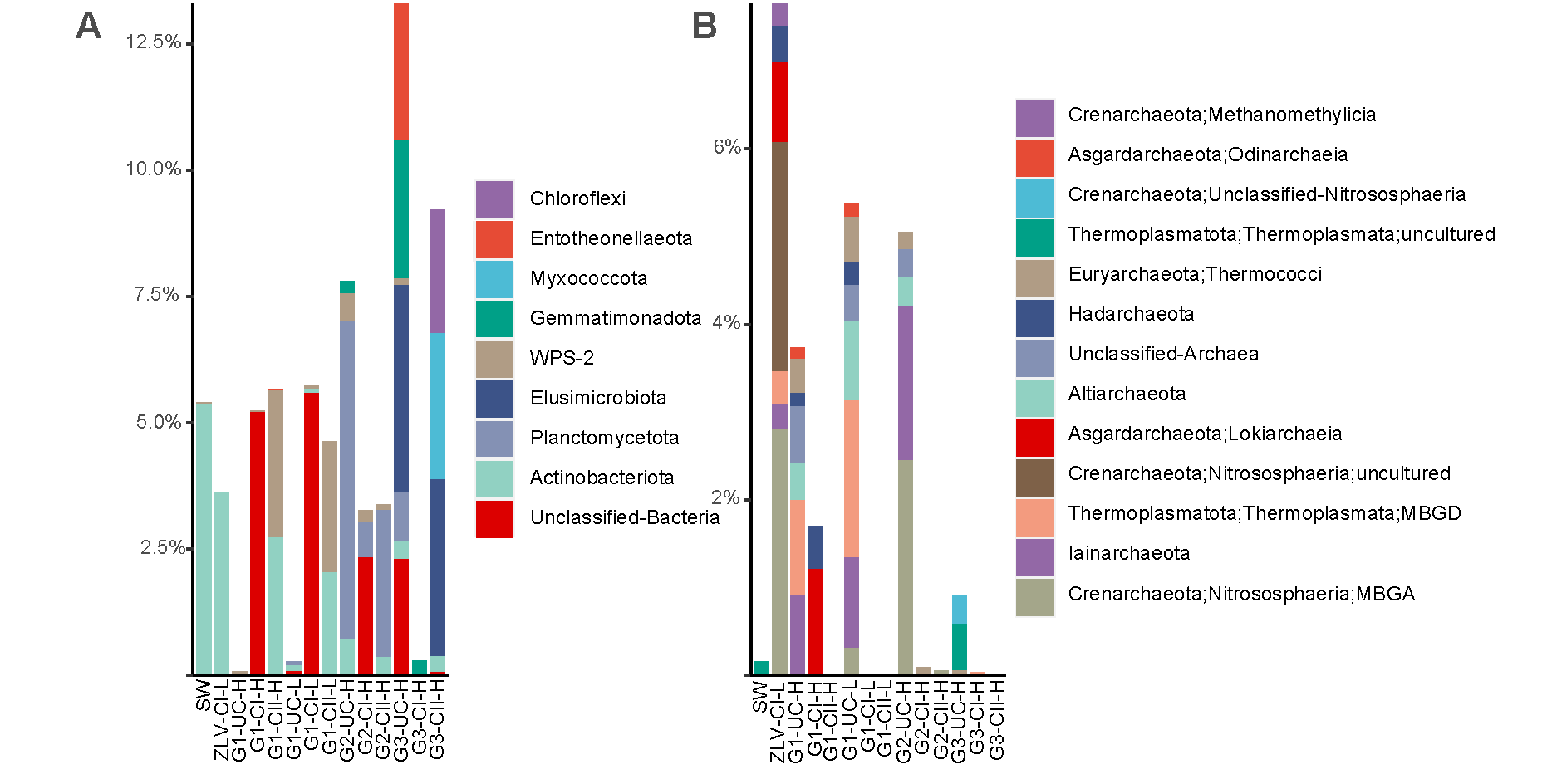


**Supplementary Figure 3**. Taxonomic compositions of bacterial (A) and archaeal (B) “others” groups. Here “others” refers to an aggregate of all taxa that did not rank in the top 10 for relative abundance. MBGD: Marine Benthic Group D and DHVEG-1. MBGA: Marine Benthic Group A.


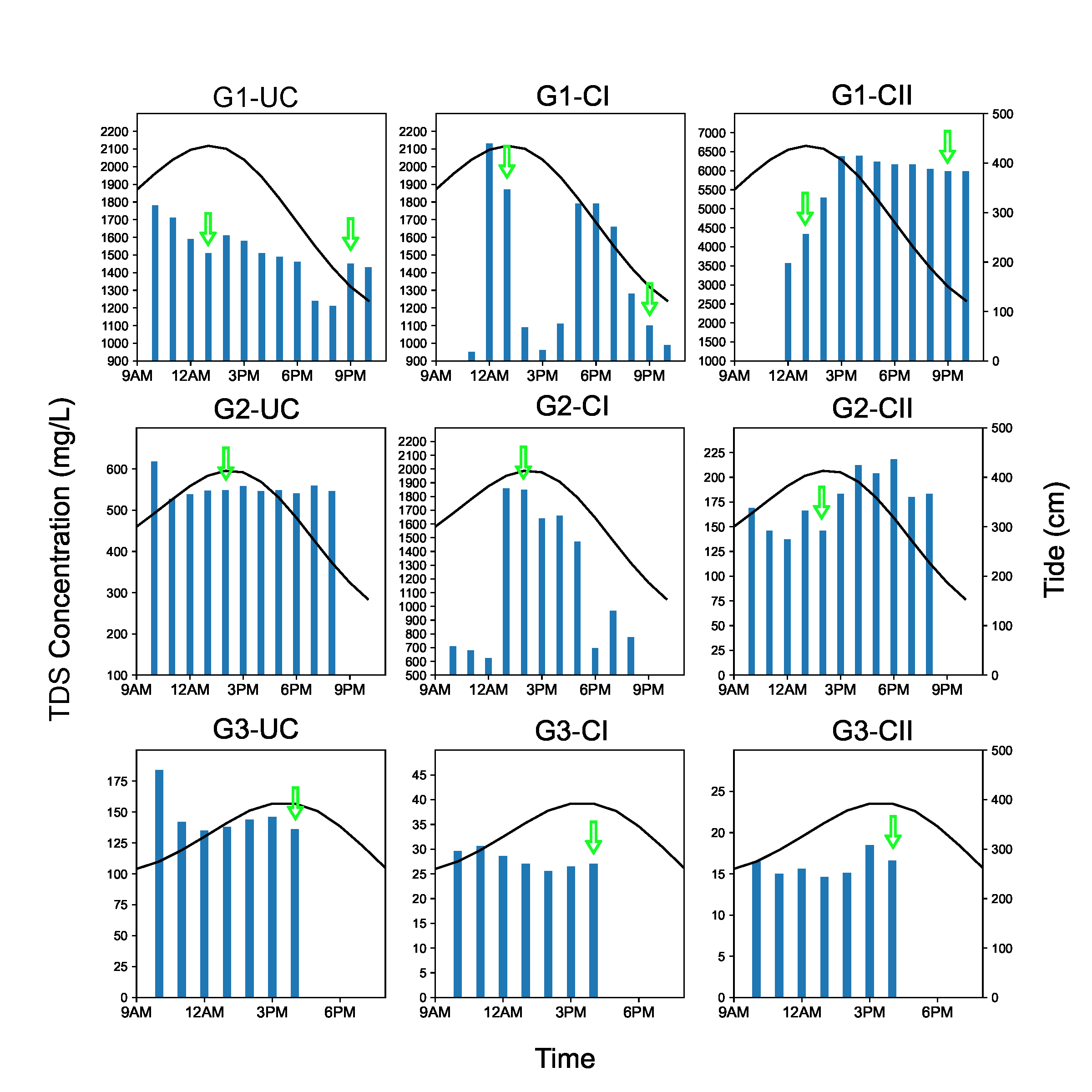


**Supplementary Figure 4**. Variation of the Total Dissolved Solids (TDS) concentrations and tide level in different underground aquifers of the three monitoring sites. Blue bars in the plot represent the TDS concentration. The black solid line above each graph indicates the tidal level of Beihai Port of Beihai City. The green arrow indicates when microbial sampling was performed. The left y-axis represents the concentrations of TDS, the right y-axis represents the tidal level, and the x-axis represents time. G1, G2 and G3 indicate monitoring site. UC, unconfined aquifer; CI, confined aquifer I; CII, confined aquifer II.





**Supplementary Figure 5**. Main groups from monitoring site G1 with a contribution to the difference between the high and low tide samples of greater than 1% based on SIMPER analysis and their relative abundance variation between high and low tide. The phylogenetic tree was constructed by the maximum likelihood method in MEGA7 (7.0) software (bootstrap: 1000). BCP: Burkholderia-Caballeronia-Paraburkholderia.


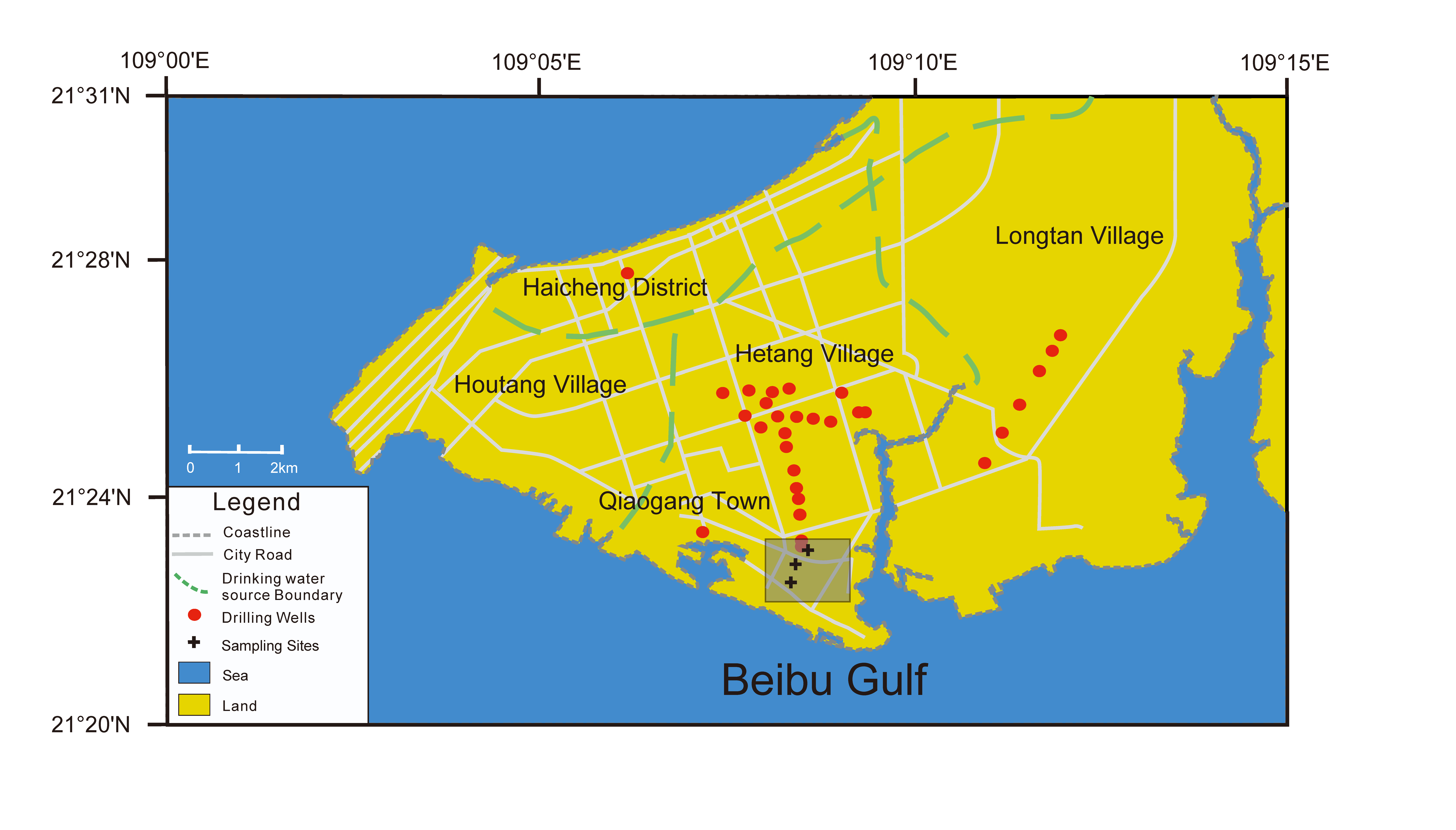


**Supplementary Figure 6**. Drinking water wells near the study area in Beihai City. Red dots represent the drinking water wells distributed within the study area and the black crosses are the locations of the three monitoring sites in this study.

**1.2 Supplementary Tables**

**Supplementary Table 3.** Microbial cell abundances in different groundwaters estimated from the 16S rRNA gene copy number

| Sample ID | Bacteria (cells/L) | Archaea (cells/L) |
| --- | --- | --- |
| SW | 2.96×10^10^ | 3.51×10^8^ |
| ZLV-CI-L | 1.28×10^7^ | 6.00×10^6^ |
| G1-UC-H | 1.30×10^10^ | 1.83×10^8^ |
| G1-CI-H | 4.88×10^10^ | 7.25×10^8^ |
| G1-CII-H | 2.77×10^8^ | 2.89×10^7^ |
| G1-UC-L | 2.92×10^10^ | 3.57×10^8^ |
| G1-CI-L | 8.19×10^10^ | 9.90×10^8^ |
| G1-CII-L | 7.35×10^8^ | 1.05×10^8^ |
| G2-UC-H | 7.19×10^7^ | 1.66×10^6^ |
| G2-CI-H | 5.58×10^9^ | 1.08×10^8^ |
| G2-CII-H | 1.18×10^8^ | 5.86×10^6^ |
| G3-UC-H | 4.02×10^8^ | 1.37×10^7^ |
| G3-CI-H | 7.52×10^7^ | 2.01×10^6^ |
| G3-CII-H | 1.87×10^8^ | 6.58×10^6^ |

**Supplementary Table 4.** α diversity indices of bacteria and archaea communities

| Sample ID | Shannon | | Chao1 | | Simpson | |
| --- | --- | --- | --- | --- | --- | --- |
|  | Bacteria | Archaea | Bacteria | Archaea | Bacteria | Archaea |
| SW | 4.94 | 2.70 | 196.00 | 145.83 | 0.89 | 0.62 |
| ZLV-CI-L | 8.28 | 5.82 | 586.00 | 231.40 | 0.99 | 0.95 |
| G1-UC-H | 6.20 | 6.20 | 289.00 | 310.75 | 0.95 | 0.94 |
| G1-CI-H | 4.13 | 1.11 | 116.00 | 48.00 | 0.87 | 0.25 |
| G1-CII-H | 4.78 | 1.90 | 153.00 | 39.00 | 0.90 | 0.47 |
| G1-UC-L | 6.47 | 6.45 | 314.00 | 372.00 | 0.95 | 0.95 |
| G1-CI-L | 3.88 | 0.65 | 125.00 | 72.00 | 0.83 | 0.14 |
| G1-CII-L | 4.63 | 2.05 | 125.00 | 39.00 | 0.89 | 0.52 |
| G2-UC-H | 8.30 | 7.34 | 611.10 | 312.00 | 0.99 | 0.99 |
| G2-CI-H | 7.86 | 2.68 | 485.00 | 44.75 | 0.99 | 0.69 |
| G2-CII-H | 7.50 | 3.39 | 423.33 | 55.00 | 0.99 | 0.82 |
| G3-UC-H | 7.65 | 3.32 | 426.00 | 96.00 | 0.99 | 0.78 |
| G3-CI-H | 7.77 | 4.36 | 446.67 | 41.00 | 0.99 | 0.93 |
| G3-CII-H | 7.45 | 3.83 | 446.25 | 54.25 | 0.99 | 0.90 |

**Supplementary Table 5.** ANOISM and PERMANOVA significance test of the different groups classified in NMDS analysis

| Bacteria | | | |  | Archaea | | | |
| --- | --- | --- | --- | --- | --- | --- | --- | --- |
|  | ANOSIM | | PERMANOVA |  |  | ANOISM | | PERMANOVA |
|  | *P* | r | *P* |  |  | *P* | r | *P* |
| Group 1 vs 2 | 0.027 | 0.92 | 0.027 |  | Group 1 vs 2 | 0.003 | 1 | 0.004 |
| Group 1 vs 3 | 0.022 | 0.67 | 0.022 |  |  |  |  |  |
| Group 2 vs 3 | 0.022 | 0.67 | 0.027 |  |  |  |  |  |
| Group 1: G1-CI-H/L, G1-CII-H/L Group 2: G1-UC-H/L, G2-UC-H, G2-CI-H Group 3: G3-UC-H, G3-CI-H, G3-CII-H, G2-CII-H | | | |  | Group 1: G1-UC-H/L, G2-UC-H Group 2: G1-CI-H/L, G1-CII-H/L, G2-CI-H  G2-CII-H, G3-UC-H, G3-CI-L, G3-CII-H | | | |
